# e-Book on plant virus infection—a cell biology perspective

**DOI:** 10.3389/fpls.2013.00203

**Published:** 2013-06-17

**Authors:** Jean-François Laliberté, Peter Moffett, Hélène Sanfaçon, Aiming Wang, Richard S. Nelson, James E. Schoelz

**Affiliations:** ^1^INRS-Institut Armand-Frappier, Institut National de la Recherche ScientifiqueLaval, QC, Canada; ^2^Département de biologie, Université de SherbrookeSherbrooke, QC, Canada; ^3^Pacific Agri-Food Research Centre, Agriculture and Agri-Food CanadaSummerland, BC, Canada; ^4^Samuel Roberts Noble Foundation, Inc.Ardmore, OK, USA; ^5^Division of Plant Sciences, University of MissouriColumbia, MO, USA

Plant viruses cause extensive remodeling of infected cells. These structural alterations include reshaping of large organelles (e.g., chloroplast, mitochondria, peroxisomes), proliferation of membranes and membranous vesicles and modification of the plasmodesmata (PD) structure. These alterations have a profound impact on plant physiology and development. However, it is only recently that studies have deeply investigated the biogenesis of virus-induced structures and their biological function(s).

Novel techniques that allow easy expression of viral proteins or modified viral genomes in plants combined with powerful visualization tools [e.g., confocal microscopy, electron microscopy (EM) tomography] offer a new perspective on cellular remodeling in virus-infected plant cells. We now know that some of these novel sub-cellular structures are virus-induced organelles or “factories” that house the RNA replication complex. Other morphological changes (e.g., membrane vesicles, alteration of the PD, cell wall-associated tubular structures) are related to the intracellular and intercellular movement of the virus. Finally, induction of autophagosomes and modification of large organelles have been observed in association with the innate immune response.

This e-Book on Plant Virus Infection—a Cell Biology Perspective aims at providing the latest information on the molecular and cellular requirements that underlie the biogenesis of these virus-induced structures.

First, we have a look at a non-virus pathogen: the viroid. In contrast to a virus, the viroid genome is composed of a tiny circular RNA (250–400 nt) that does not code for proteins. Nevertheless, viroid infections are accompanied by cellular and developmental disorders that sometimes have dramatic economic consequences, for instance the cadang-cadang disease of coconut palms. Di Serio et al. (2013) look at the cytopathic effects incited by viroid RNAs and propose mechanisms that may lead to these morphological changes.

“Hijack it, change it” is applicable to the description of many viral processes, given the propensity of viruses to commandeer the host machinery for their own purposes. In their review, Patarroyo et al. (2013) discuss this concept as applied to the secretory system. They provide an overview of the plant secretory pathway and discuss recent advances in our understanding of how viruses utilize and alter this system in order to permit such processes as replication, intra- and inter-cellular movement.

Sanfaçon (2013) summarizes the membrane-localization characteristics of proteins encoded by members of the *Secoviridae*, She further reviews and discusses literature demonstrating the ability of these proteins to induce membrane proliferation and perhaps alter membrane structure (e.g., curvature) and integrity (formation of pores through oligomerization) in the host cell. The membrane often targeted by these viral proteins is the endoplasmic reticulum.

In reviewing the latest cell biological studies describing tobacco mosaic virus (TMV) replication and movement, Liu and Nelson (2013) highlight how a divergent function of two orthologous proteins may explain different infection requirements by two tobamoviruses. The authors review the many publications showing influences or interactions of proteins encoded by TMV as infections develop. The complexity of the interactions and their proposed functions for just this one virus are striking. The findings from multiple laboratories strongly suggest that the host membrane is a primary vehicle for tobamovirus intercellular movement and that the cytoskeleton has a role in modulating movement for only some members. A theory that cytoskeletal-dependent trafficking of a TMV complex from the PD is necessary for TMV, but not the related *Turnip vein clearing virus* is presented.

The triple gene block (TBG) proteins are the focus of several contributions. First, Solovyev et al. (2012) introduce the reader to the complex story of viral cell-to-cell movement mediated by TGB proteins of potex- and hordei-like viruses. The authors provide links between virus cell-to-cell trafficking and replication, silencing suppression, virus systemic spread over the plant, and the roles of the nucleus in plant virus movement.

Indeed, TGB proteins may have multiple functions in the viral infection process. Linnik et al. (2013) contribute a research article on how three-dimensional structured illumination microscopy (3D-SIM) can provide a novel high definition view of the potato virus X (PVX) factory architecture. They show previously unrecognized membrane structures induced by the PVX TGB proteins. Specifically, they found that previously observed granular structures produced by TGB2 and TGB3 proteins are fine membrane doughnut-shaped loops of remodeled tubular ER containing the viral proteins. These loops form dense arrays wrapped around the TGB1 protein inclusions. These findings provide new insights into the structural organization of a PVX complex considered as a virus factory.

Interestingly, a second research paper studying a different *Potexvirus* genus member, alternanthera mosaic virus (AltMV), provides insight into how its TGB3 protein may function to modify its target, the chloroplast and its membranes. In this study, Jang et al. (2013) further solidified a previously observed influence of AltMV TGB3 protein on chloroplast structure through EM studies. They also found that TGB3 interacts with a nuclear-encoded chloroplast protein that may result in a destabilization of the thylakoid membranes. While shedding light on an influence of a viral protein on another membrane system, this and the previous study on PVX indicate how conserved proteins may have diverged in function.

In addition to TGB3, TGB2 seems also to play a role in targeting the virus to chloroplasts. In another research paper, Cowan et al. (2012) demonstrated that TGB2 protein of potato mop-top virus (PTMV), the type virus of the genus *Pomovirus*, interacts with the ER, mobile granules, small round structures, and chloroplast envelops. Protein-lipid interaction assays confirmed the association of TGB2 with lipids of chloroplasts. Consistently, EM data revealed abnormal chloroplasts with cytoplasmic inclusions and terminal projections. Viral coat protein (CP), genomic RNA, and labeled TGB2 were colocalized to chloroplasts in PTMV-infected tissues.

Viral cell-to-cell movement remains a hot topic for plant virologists. Xu and Zhou (2012) studied NSvc4, the movement protein of rice stripe virus (RSV), the type member of the *Tenunivirus* genus. They showed that NSvc4 traffics on the actin filament and ER network and that targeting of NSvc4 to PD requires the functional cytoskeleton. They also found that NSvc4 contains a chloroplast-targeting signal and localizes to chloroplasts in infected cells, suggesting NSvc4 may also be a multi-functional protein.

In their hypothesis and theory article, Krenz et al. (2012) discuss alteration in chloroplast structure induced by geminiviruses. They provide evidence that abutilon mosaic virus (AbMV) infection induces a network of stromules that extend from the plastid to the cellular periphery. The stromules contain heat shock cognate 70 kDa protein, a plant chaperone that interacts with the AbMV movement protein. The authors discuss a model in which AbMV traffics along the stromule network to move into neighboring cells.

Cellular remodeling is also the consequence of molecular pathways being overpowered by viruses. Verchot (2012) explores the recruitment of host proteins, such as cellular chaperones, to membrane bound sites required for virus replication and cell-to-cell movement. She discusses the possibilities that cellular chaperones are acting within their normal context to enable viral protein folding, trafficking, and functioning, or whether they are diverted from their normal activities to provide novel contributions to virus infection.

Zhang and Wang (2012) summarize current knowledge about the unfolded protein response (UPR) in cells, which is a reaction to ER stress triggered by the accumulation of unfolded or misfolded proteins in the lumen of the endoplasmic reticulum. The UPR is an attempt by the cell to return to ER homeostasis. Both animal and plant viruses are capable of redirecting the cell to produce large amounts of viral proteins, which causes ER stress and sets in motion the UPR signaling pathways. Zhang and Wang (2012) discuss how viral infections activate the UPR and implications for host physiology.

Bujarski (2013) gives an overview of genetic recombination in plant positive-sense RNA viruses. Questions being raised are the identity of the host factors involved in RNA recombination and the intracellular location of this RNA–RNA template switching.

## References

[B1] Bujarski J. J. (2013). Genetic recombination in plant-infecting messenger-sense RNA viruses: overview and research perspectives. Front. Plant Sci. 4:68 10.3389/fpls.2013.00068 23533000 PMC3607795

[B2] Cowan G. H. Roberts A. G. Chapman S. N. Ziegler A. Savenkov E. I. Torrance L. (2012). The potato mop-top virus TGB2 protein and viral RNA associate with chloroplasts and viral infection induces inclusions in the plastids. Front. Plant Sci. 3:290 10.3389/fpls.2012.00290 23269927 PMC3529358

[B3] Di Serio F. De Stradis A. Delgado S. Flores R. Navarro B. (2013). Cytopathic effects incited by viroid RNAs and putative underlying mechanisms. Front. Plant Sci. 3:288 10.3389/fpls.2012.00288 23308076 PMC3538276

[B4] Jang C. Seo E.-Y. Nam J. Bae H. Gim Y. G. Kim H. G. (2013). Insights into Alternanthera mosaic virus TGB3 functions: interactions with Nicotiana benthamiana PsbO correlate with chloroplast vesiculation and veinal necrosis caused by TGB3 overexpression. Front. Plant Sci. 4:5 10.3389/fpls.2013.00005 23386854 PMC3560364

[B5] Krenz B. Jeske H. Kleinow T. (2012). The induction of stromule formation by a plant DNA-virus in epidermal leaf tissues suggests a novel intra- and intercellular macromolecular trafficking route. Front. Plant Sci. 3:291 10.3389/fpls.2012.00291 23293643 PMC3530832

[B6] Linnik O. Liesche J. Tilsner J. Oparka K. J. (2013). Unraveling the structure of viral replication complexes at super-resolution. Front. Plant Sci. 4:6 10.3389/fpls.2013.00006 23386855 PMC3560349

[B7] Liu C. Nelson R. S. (2013). The cell biology of Tobacco mosaic virus replication and movement. Front. Plant Sci. 4:12 10.3389/fpls.2013.00012 23403525 PMC3568708

[B8] Patarroyo C. Laliberté J.-F. Zheng H. (2013). Hijack it, change it: how do plant viruses utilize the host secretory pathway for efficient viral replication and spread? Front. Plant Sci. 3:308 10.3389/fpls.2012.00308 23335933 PMC3542527

[B9] Sanfaçon H. (2013). Investigating the role of viral integral membrane proteins in promoting the assembly of nepovirus and comovirus replication factories. Front. Plant Sci. 3:313 10.3389/fpls.2012.00313 23439982 PMC3557413

[B10] Solovyev A. G. Kalinina N. O. Morozov S. Y. (2012). Recent advances in research of plant virus movement mediated by triple gene block. Front. Plant Sci. 3:276 10.3389/fpls.2012.00276 23248633 PMC3520053

[B11] Verchot J. (2012). Cellular chaperones and folding enzymes are vital contributors to membrane bound replication and movement complexes during plant RNA virus infection. Front. Plant Sci. 3:275 10.3389/fpls.2012.00275 23230447 PMC3515963

[B12] Xu Y. Zhou X. (2012). Role of rice stripe virus NSvc4 in cell-to-cell movement and symptom development in *Nicotiana benthamiana*. Front. Plant Sci. 3:269 10.3389/fpls.2012.00269 23233857 PMC3516811

[B13] Zhang L. Wang A. (2012). Virus-induced ER stress and the unfolded protein response. Front. Plant Sci. 3:293 10.3389/fpls.2012.00293 23293645 PMC3531707

